# Innate and Adaptive Cell Populations Driving Inflammation in Dry Eye Disease

**DOI:** 10.1155/2018/2532314

**Published:** 2018-08-12

**Authors:** José L. Reyes, Danielle T. Vannan, Bertus Eksteen, Imelda Juárez Avelar, Tonathiu Rodríguez, Marisol Ibet González, Alicia Vázquez Mendoza

**Affiliations:** ^1^Universidad Nacional Autónoma de México (UNAM), Laboratorio de Inmunología experimental y Regulación de la inflamación Hepato-Intestinal, UBIMED, Facultad de Estudios Superiores (FES) Iztacala, Tlalnepantla, MEX, Mexico; ^2^University of Calgary, Snyder Institute for Chronic Diseases, Cumming School of Medicine, Calgary, AB, Canada; ^3^Aspen Woods Clinic, Calgary, AB, Canada; ^4^Universidad Nacional Autónoma de México (UNAM), Unidad de Biomedicina (UBIMED), Facultad de Estudios Superiores (FES) Iztacala, Tlalnepantla, MEX, Mexico; ^5^Universidad Nacional Autónoma de México (UNAM), Laboratorio de Enfermedades Inflamatorias Oculares, Carrera de Optometría, Facultad de Estudios Superiores (FES) Iztacala, Tlalnepantla, MEX, Mexico

## Abstract

Dry eye disease (DED) is the most common ocular disease and affects millions of individuals worldwide. DED encompasses a heterogeneous group of diseases that can be generally divided into two forms including aqueous-deficient and evaporative DED. Evidence suggests that these conditions arise from either failure of lacrimal gland secretion or low tear film quality. In its secondary form, DED is often associated with autoimmune diseases such as Sjögren's syndrome and rheumatoid arthritis. Current treatment strategies for DED are limited to anti-inflammatory medications that target the immune system as the source of deleterious inflammation and tissue injury. However, there is a lack of understanding of the underlying pathogenesis of DED, and subsequently, there are very few effective treatment strategies. The gap in our knowledge of the etiology of primary DED is in part because the majority of research in DED focused on secondary autoimmune causes. This review focuses on what is currently understood about the contribution of innate and adaptive immune cell populations in the pathogenesis of DED and highlights the need to continue investigating the central role of immunity driving DED.

## 1. Introduction

### 1.1. Definition and Diagnosis of DED

Dry eye disease (DED) is a multifactorial condition involving the ocular surface, lacrimal glands, and meibomian glands leading to abnormal tear film quantity and/or quality. Characterized by discomfort and visual disturbance, DED may lead to loss of vision caused by exposure of the ocular surface to excessive desiccant stress. Due to the high prevalence of DED worldwide, it is a critical public health issue [1–3].

The Dry Eye Workshop (DEWS) has divided DED into two major classes: aqueous tear-deficient dry eye disease and evaporative dry eye disease. The former is often associated with autoimmune Sjögren's syndrome (ssDED) and is characterized by dysfunction of both lacrimal and salivary glands resulting in reduced tear secretion rate and/or volume. In evaporative DED, there is excessive fluid loss from the exposed ocular surface in the presence of normal lacrimal secretory function. The development of evaporative DED has also been associated with intrinsic factors such as meibomian oil deficiency, disorders of the eyelid, and low blink rate. Extrinsic factors that can also influence DED onset include vitamin A deficiency, topical drug preservatives, contact lens wearing, certain prescription drugs, and seasonal allergies [4].

Accurate diagnosis of DED requires completion of the ocular surface disease index (OSDI) questionnaire that addresses potential risk factors as well as clinical tests to assess tear production and elimination, tear stability, and ocular surface integrity [5, 6]. Advanced tools including confocal microscopy, vision function, and conjunctival cytology can also be applied to improve diagnostic accuracy of DED [7–9].

### 1.2. Dry Eye Disease Immunobiology

Studies have described certain immunomodulating factors involved in maintenance of the ocular surface that may be disrupted during DED. Recently, it was identified that retinoic acid, a metabolite of vitamin A, is critical for induction of Foxp3^+^ T regulatory cells and contributes to the immune privilege of the eye [10]. Additionally, tear hyperosmolarity, hormonal changes, and mechanical irritation can also contribute to the onset of DED pathology [11–14]. Studies have shown that an imbalance of tear film components such as higher concentrations of sodium can result in increased osmolarity of the tear film and lead to inflammation with the potential to damage the ocular surface in part due to goblet cell apoptosis [15–17]. Persistent hyperosmolarity is further sustained by increased concentration of inflammatory cytokines and matrix metalloproteinases (MMPs) [18].

Therefore, inflammation associated with the eye has attracted interest from researchers worldwide in an effort to understand the immunological processes associated with the development of DED.

As in many chronic diseases, sustained or dysregulated inflammation consisting of increased proinflammatory cytokine levels and infiltration of immune cells has been identified. Therefore, targeting immune cells or inflammatory mediators may have therapeutic potential. Unfortunately, little is known about the specific pathogenic cell populations in DED.

In this review, unless stated otherwise, we are indicating DED as independent from autoimmune Sjögren's syndrome. We have summarized the available evidence for the role of both innate and adaptive cell populations as well as cytokines, chemokines, and their respective receptors in the pathology of DED.

## 2. Search Methodology

The following keywords were used to search the Pubmed database: dry eye disease, desiccant stress, ocular surface inflammation, and non-autoimmune. Papers published from 2000 to 2017 were reviewed. Limited focus was placed on Sjögren's syndrome-associated DED since there are already excellent reviews published [19, 20].

## 3. Innate Immunity Is a Driving Force in the Pathogenesis of DED

The innate inflammatory response is required to eliminate potential harmful pathogens and can contribute to tissue remodeling after injury. However, if dysregulated, this type of immune response may lead to sustained inflammation resulting in compromised host organ functions. Thus, understanding the inflammatory pathways activated during DED may provide insight to potential therapeutic targets.

### 3.1. Neutrophils

Neutrophils are short-lived polymorphonuclear cells (PMNs) predominantly associated with frontline resistance against pathogens. In addition to their ability to phagocytize potentially harmful antigens, neutrophils can activate potent antimicrobial defense mechanisms such as the release of reactive oxygen species (ROS) and extracellular neutrophilic DNA traps (NETs) [21–23].

**Figure 1:**
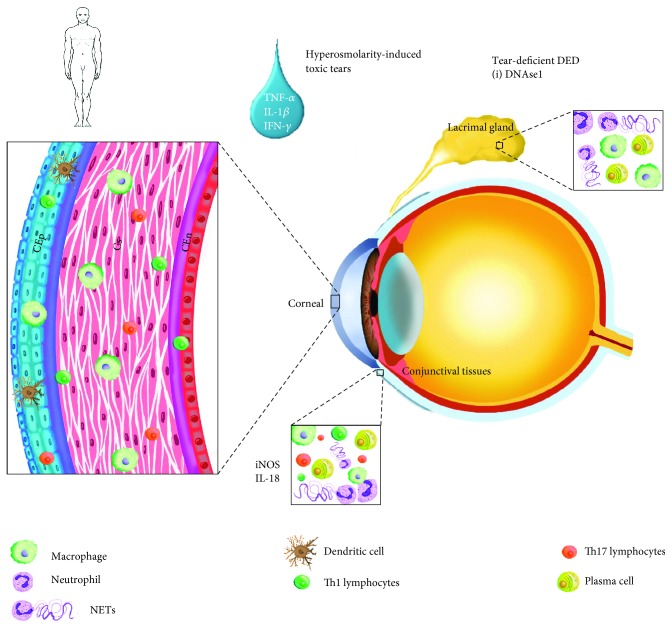
Schematic view of humoral and cellular inflammatory components found increased in patients with DED. Abbreviations: En: corneal endothelium; CEp: corneal epithelium; Cs: corneal stroma; IFN: interferon; iNOS: inducible nitric oxide synthase; NETs: neutrophil-derived extracellular traps; TNF: tumor necrosis factor.

Neutrophils have been detected on the ocular surface in patients with DED and are often colocalized with histone, neutrophil elastase, and extracellular DNA (eDNA) consistent with NET release. However, whether their presence is pathogenic or regulatory remains untested [24]. A recent study evaluating the presence of DNAse 1 in the lacrimal gland compared between a cohort of patients with symptomatic, tear-deficient DED, and asymptomatic individuals with normal tear production. The authors observed a significant decrease in the concentration of nucleases present in the tear fluid from patients with DED compared to healthy individuals. In addition, patients with DED had greater accumulation of eDNA on their ocular surface compared to the healthy controls. It is possible that the lack of nucleases and accumulation of eDNA and NETs on the precorneal surface may contribute to ocular surface inflammation in these patients (see Figure 1). This report is the first to suggest that by-products of neutrophil activation may contribute to DED in patients; however, further investigation is required to confirm these observations [24].

Previously, it has been established that tear hyperosmolarity can trigger DED; however, whether this alters neutrophil responses is not completely understood. Tibrewal et al. reported that hyperosmolar stress, generated by increasing concentration of NaCl, induced NETosis compared to cells incubated in isoosmolar media. Furthermore, neutrophils exposed to hyperosmolar media exhibited morphological changes such as larger and rounded nuclei occupying the majority of the cell. In order to confirm the impact of hyperosmolar conditions on NET formation, the authors restored isoosmolar conditions, and subsequently, NET formation decreased. Furthermore, the addition of NET formation inhibitors (i.e., staurosporine and anti-*β*2 integrin) resulted in lower numbers of NETs under hyperosmolar conditions. An association between hyperosmolarity and NET formation has been demonstrated and suggests that there may be a potential benefit of NET inhibition during DED [25].

**Figure 2:**
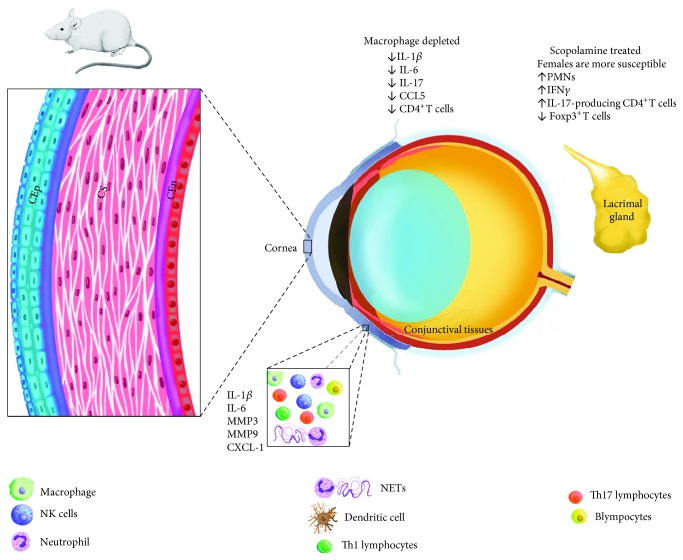
Schematic view of humoral and cellular inflammatory components found in experimental DED. Abbreviations: CEn: corneal endothelium; CEp: corneal epithelium; Cs: corneal stroma; IFN: interferon; iNOS: inducible nitricoxide synthase; MMP: matrix metalloprotease; NETs: neutrophil-derived extracellular traps; PMN: plymorphonuclear; TNF: tumor necrosis factor.

Neutrophils are known for eliciting a robust immune response by responding to chemokines and inducing the release of proinflammatory mediators. In a mouse model of DED involving corneal damage by alkali burn, an increased production of inflammatory mediators such as interleukin (IL)-1*β*, IL-6, and matrix metalloproteinases (MMPs) and CXCL1 chemokine in whole corneal homogenates was observed. Analysis of myeloperoxidase (MPO) activity in corneal lysates suggested a significant increase in neutrophils (see Figure 2). When the mice were topically treated with either dexamethasone (Dex) or doxycycline (Doxy), there was a reduction in tissue injury and inflammatory mediators. While both treatments attenuated the degree of inflammation, a differential effect was observed, that is, Dex treatment significantly decreased IL-6; MMPs −1, −3, −9, and −13; and TIMP-1 but enhanced MMP-8 transcripts up to 1000-fold. In contrast, Doxy treatment showed greater suppression of IL-6, MMP-8, MMP-9, and MMP-13. Moreover, Dex-treated corneas had a significant decrease in Gr1^+^ cell counts as compared to vehicle-treated corneas at days two and five postinjury. Therefore, corneal opacity was associated with neutrophil infiltration, most likely, CXCL-1 mediated [26].

Sex-associated changes in neutrophils influencing the susceptibility to DED have also been identified in mouse models. Inhibiting lacrimal gland function using scopolamine in female mice resulted in more severe DED and lower numbers of lipoxin A4- (LXA4-) producing PMNs in the corneal limbus, lacrimal gland, and cervical lymph nodes compared to male mice. This also correlated with heightened expansion of IFN-*γ*- and IL-17-producing CD4^+^ T cells and a significant decrease in Foxp3^+^ regulatory T cells (see Figure 2). Additionally, when neutrophils were depleted by intraperitoneally delivered anti-Ly6G antibodies, levels of LXA4 decreased concomitantly with uncontrolled proliferation of Th1 and Th17 CD4^+^ cells. Interestingly, topical and systemic administration of LXA4 restored numbers of Foxp3^+^ regulatory T cells and drastically suppressed Th1 cells and to a lesser degree Th17 cells resulting in attenuated DED. Thus, a previously unknown sex-specific, T regulatory-promoting function via LXA4 release in neutrophils was uncovered [27].

Sjögren's syndrome is an autoimmune-mediated eye pathology where one of the main identified autoantigens is the 48KDa ribonucleoprotein, also known as antigen B. Interestingly, antigen B is expressed in the nucleus and surface membrane of human neutrophils which is released when neutrophils undergo apoptosis. Whether targeting neutrophils worsens disease outcome has not been explored; however, it has been shown that antigen B activates the MAP kinase pathway and induces IL-8 output from a donor neutrophil and a neutrophil cell line [28].

Growing evidence demonstrates that neutrophil infiltration occurs in patients with DED as well as in experimental murine models with potentially deleterious outcomes. To date, a role for early infiltrating neutrophils has been shown. However, further research is required to clarify if early versus late cellular recruitment of neutrophils can drive differential function within the tissue. Understanding more about the neutrophil phenotypes similar to other innate immune cells like macrophages is required to direct future development of therapeutic targets.

### 3.2. Macrophages

Macrophages are innate immune cells with a wide variety of functions ranging from recognition and clearance of pathogens to activation and polarization of adaptive immune cells. Other macrophage features include tissue repair and secretion of growth factors promoting angiogenesis [29]. Macrophages are highly plastic cells as evidenced by differential responses exerted by these cells depending on encountering either Th1- (IFN-*γ-*) or Th2- (IL-4-) dominated environments. Macrophages contribute to the spectrum of immune responses by acquiring polarized phenotypes that can be broadly classified as classically activated (M1) or alternatively activated (M2) macrophages [30, 31]. The role of macrophages has recently been addressed in DED particularly associated with immune-mediated eye injury.

In Sjögren's syndrome, both lacrimal and salivary glands can become severely damaged. Manoussakis et al. found that when comparing patients with DED, macrophage infiltration in the periepithelial area was greater in samples from patients with Sjögren's syndrome than patients without. In contrast, dendritic cell (DC) distribution was mostly intraepithelial with consistently higher numbers in patients with Sjögren's syndrome. Interestingly, periepithelial CD68^+^ macrophages were found to also be positive for IL-18, which positively correlated with glandular inflammation [32]. In a separate study, minor salivary gland (MSG) biopsies from patients with primary Sjögren's syndrome (pSS) had increased numbers of CD68^+^ macrophages that correlated with the grade of inflammation and neovascularization [33]. Therefore, in the case of autoimmune Sjögren's syndrome, there appears to be a role for macrophages to infiltrate the ocular tissue and contribute to eye inflammation.

Due to the plastic nature of macrophages, You et al. evaluated the presence and polarity of macrophages during desiccant stress-induced DED in the cornea and conjunctival epithelium. The authors did not detect any significant changes in the number of macrophages over time in either the cornea or conjunctiva. Measurement of canonical markers of macrophage activation in conjunctival tissue demonstrated that M1-associated marker iNOS expression was increased and paralleled IL-18 high levels on day 10 postinjury; however, arginase-1 remained unchanged. This data suggests that there may be a time-dependent effect on macrophage phenotypes in DED [34]. In line with this, others have also reported M1-like cells in the salivary gland during experimental Sjögren's syndrome [35]. However, care should be taken in interpreting differences in macrophages during DED as Zhou et al. have shown increased macrophage infiltration in the corneal limbus in AIRE-deficient mice, a systemic autoimmune model affecting the lacrimal glands. They reported substantial infiltration of macrophages into the cornea, and when depleted with locally delivered clodronate-loaded liposomes, corneal tissue thickness and opacity were attenuated. Others have also reported that macrophage depletion attenuates experimental models of DED predominantly by decreasing inflammatory mediators (e.g., IL-1*β*, IL-6, IL-17, and CCL5) and CD4^+^ T cells [36]. Taken together, this data suggests that macrophages likely contribute to inflammation present in autoimmune-mediated DED whereas the data is not as consistent for nonautoimmune cases of DED [37].

Changes in circulating monocytes have also been observed during DED. In a report by Hauk et al., they showed that CD14^+^ monocytes isolated from peripheral blood in patients with pSS had reduced ability to phagocytize apoptotic salivary gland epithelial cells compared to monocytes from healthy donors. The monocytes exhibited heightened expression of vasoactive intestinal peptide (VIP) receptor 2 (VPAC2) but not VPAC1. However, when testing monocyte response to VIP, neither phagocytic ability nor inflammatory mediator release (TNF-*α*) was altered in the presence of recombinant VIP [38]. Authors highlight the fact that VPAC2 overexpression in monocytes might reflect compromised monocyte features upon apoptotic cell clearance (i.e., aberrant secretion of TNF-*α* rather than TGF*β* release).

### 3.3. NK Cells

Natural killer (NK) cells are a group of innate lymphocytes with cytotoxic function including secretion of membrane-disrupting granzyme and perforin largely aimed at clearing transformed and infected host cells [39–41]. Furthermore, they have proven to influence the immune response by secreting large amounts of cytokines, mainly IFN-*γ*, resulting in activation of neighboring cell populations such as macrophages and T cells. NK cells have been widely studied under a variety of pathologies ranging from infectious to autoimmune [42–45]. Although the role of NK cells has been explored in Sjögren's syndrome, little is known in regard to their role in DED outside of an autoimmune disease context. A cohort of 106 patients with DED was evaluated to determine the expression of inhibitory receptors for NK cells known as KIRs. It was found that the inhibitory NK cell receptor KIR2DS2 in combination with HLA-C1 allele, but not other KIRs, was frequently associated with patients suffering from severe DED compared to healthy donors suggesting an underlying genetic link between NK cells and DED susceptibility in patients [46]. In a pioneering study, Chen et al. reported an early increase in NK1.1 transcript expression in conjunctival samples from chemically induced DED in mice that was later confirmed by flow cytometry ruling out the contribution from NKT cells (NK1.1^+^ TCR*β*^−^). The percentage of conjunctival NK cells was three-fold higher in mice with DED than control mice (see Figure 2). In contrast, there was no difference in the number of NK cells present in draining lymph nodes between mice with DED and control mice; however, the number of IFN-*γ*-producing NK cells isolated from the lymph nodes was higher in DED mice as compared to control mice. Authors also found that neither NK cell depletion nor IFN-*γ* immunoneutralization correlated with lower corneal surface injury and decreased levels of TNF-*α* and IFN-*γ* cytokines in mice with DED. Interestingly, mice lacking NK cells exhibited lower levels of costimulatory molecules (CD80, CD86, and MHCII) on APCs. In the context of nonautoimmune DED, NK cell conjunctival infiltration and disease promotion are possibly driven by IFN-*γ* secretion and ultimately impact APC maturation and disease outcome [47].

A further role for NK cells contributing to DED was described by Zhang et al. Intraepithelial NK cells were found to reside in the conjunctival tissue and are further expanded upon desiccant stress caused by scopolamine administration and airflow exposure (see Figure 2). Subsequent depletion of NK cells results in lower numbers of IL-17A-producing CD4^+^ T cells in the ocular surface and cervical lymph nodes five days after desiccant stress exposure. Furthermore, in the absence of NK cells, authors noticed reduced expansion of CD11b^+^MHCII^+^ and CD11c^+^MHCII^+^ antigen-presenting cells (APCs). In order to confirm a central role for NK cells in Th17 expansion, Th17 cells from the spleen and cervical lymph nodes were harvested and transferred into nude mice depleted of NK cells, which later were protected against disruption of the corneal barrier. In contrast, the transfer of Th17 cells in the presence of intact NK cells developed ocular surface injury in mice. This report highlights the important role for NK cells in modulating APC activity leading to polarization of pathogenic Th17 cells involved in ocular surface inflammation [48].

## 4. Adaptive Immunity in DED

### 4.1. Conventional CD4^+^ T Lymphocytes

T cells and antibody-producing B cells are the main constituent cells of the adaptive immune system. T lymphocytes can be further divided in numerous subsets including the two best characterized subpopulations CD4^+^ T helper cells and cytotoxic CD8^+^ T cells. As such, these cells are considered drivers and effectors in ongoing immune responses. Although their role is central in resistance to pathogens, both CD4^+^ and CD8^+^ T cells have been implicated in numerous autoimmune and autoinflammatory diseases. A critical role for subsets of CD4^+^ T cells has been described in DED as well as Sjögren's syndrome.

The recruitment of T lymphocytes into inflamed conjunctival tissue from patients with moderate to severe DED has been characterized. Although the number of infiltrating CD4^+^ and CD8^+^ T cells was similar between patients with as Sjögren's syndrome DED and nonautoimmune DED, markers of T cell recruitment and activation were upregulated only in patients with nonautoimmune DED. In addition, ICAM expression was upregulated in conjunctival epithelial cells. This data suggests an important role for CD4^+^ T cells and integrins contributing to peripheral T cell migration and proliferation in patients with DED [15].

Ethnicity may also play a role in the differences between T lymphocytes in autoimmune and nonautoimmune DED. In a cohort of patients from Korea, authors reported a more severe phenotype of Sjögren's syndrome DED patients as compared to nonautoimmune DED patients [49]. In addition, the patients had increased CXCL11 protein levels in tear samples and CD4^+^CXCR3^+^ Th1 lymphocytes assayed by flow cytometry in conjunctival tissue. Thus, despite of differences in the autoimmune or nonautoimmune origin of DED, reports consistently demonstrate abundant infiltrating CD4^+^ T cells in ocular tissue suggesting that T cell infiltration might be a requisite to maintain the inflammatory process observed in DED.

An extensive analysis of T cells in conjunctiva samples from healthy individuals and patients with DED in Singapore was recently conducted. Bose et al. characterized T cells from healthy donors by collecting impression cytology samples (noninvasive biopsy of the conjunctival surface) and identified a clear CD8^+^ T cell dominance over CD4^+^ T cells. In addition, healthy conjunctivae were found to contain a resident population of CD8^+^/CD69^+^/CD103^+^/CCR7^−^ T lymphocytes. Using an array of T cell-specific surface markers, authors further characterized different T cell subsets present on the ocular surface from 52 patients with DED. Using these markers, patients were able to be clustered into different categories. Cluster 1 DED patients had high ocular redness and increased conjunctival CD8^+^ (T_Central Memory_) cells whereas cluster 2 DED patients had increased tear instability and higher proportions on conjunctival CD4^+^ (T_Effector Memory_) cells. Interestingly, most of the patients were grouped in cluster 2; however, when grouped based on DED subtype (i.e., aqueous deficient, evaporative, or mixed), 21% of patients did not fall into either cluster due to a unique DED phenotype with unaltered canonical DED tests (Schirmer's and break up time test) [50]. Thus, it was shown that the complex nature of DED may lead to a redistribution of T cell populations compared to T cells from samples of healthy ocular tissue.

Murine models of DED including the scopolamine-induced desiccant stress model have been employed to study the relationship between T lymphocytes and ocular inflammation. Niederkorn et al. reported that the transfer of CD4^+^ T cells from the spleen and lymph nodes of sensitized mice into nude mice resulted in a strong ocular-specific inflammatory response characterized by inflammatory foci in the cornea, conjunctiva, and lacrimal gland along with a significant decrease in the number of goblet cells and tear volume. Transfer of CD4^+^ T cells elicited inflammation on both the ocular surface and lacrimal gland even in the absence of airflow and desiccant stress. This study demonstrated that ocular surface-specific T cells are rapidly generated upon scopolamine sensitization and have the ability to induce DED in mice lacking T cells, suggesting the emergence of autoreactive CD4^+^ T cells. In addition, authors found that cotransfer of CD25^+^ T regulatory cells lead to a protective effect whereas this protection was lost upon depletion of Tregs during the transfer. The protective effect of T regulatory cells was also associated with a reduction in neutrophil infiltration [51].

### 4.2. CD4^+^ Th17 Lymphocytes

A tremendous focus has been placed on the Th17 response in numerous inflammatory diseases [52–54]. In the case of DED, De Paiva et al. observed a strong expression of the IL-23/Th17 axis including the Th17-promoting cytokines IL-6, IL-23R, TGF*β*2, and ROR*γ*t transcription factor (expressed by committed Th17 cells) in DED patients. In confirmation, using the scopolamine-induced DED mouse model, authors found increased levels of IL-17 in tear samples from mice with DED and abundant ocular surface IL-17-producing cells. Neutralization of IL-17 led to an attenuation of DED in mice; however, the cellular source of IL-17 was not conclusively determined [55].

More recently, the presence of Th17 cells and their role in driving DED were addressed by means of chemically induced experimental DED. Dohlman et al. demonstrated significant expansion of IL-17-secreting CD4^+^ T cells rather than Th1 (IFN-*γ*-secreting T cells) in draining lymph nodes from animals receiving desiccant stress for 12 days. In addition, authors confirmed the presence of CCR6 chemokine receptor on the surface membrane of IL-17-secreting CD4^+^ T cells. The authors further quantified the expression of the CCR6 ligand CCL20 on corneal and conjunctiva tissue. Functional assays aimed to test the importance of the CCR6-CCL20 axis in DED were conducted by immune neutralization of subconjunctival-delivered blocking anti-CCL20 antibody which resulted in reduced numbers of Th17 cells in both draining lymph nodes and conjunctival samples. Moreover, this later correlated with improved clinical signs in DED mice (reduced corneal epitheliopathy) and decreased mRNA expression of inflammatory mediators such as IL-6, MMP3, and IFN-*γ* in corneal and conjunctival tissues [56]. The impact of Th1- and Th17-specific chemokine receptors, CXCR3 and CCR6, respectively, in DED outcome was also measured. The authors found a high percent of CCR6^+^CD4^+^ T cells in both cervical lymph nodes and the ocular surface five days after desiccant stress induction whereas CXCR3^+^CD4^+^ T cells are seen increased only on the ocular surface. The role of these CCR6 and CXCR3 chemokine receptors was tested in mice lacking either receptor. Unlike wild-type mice that exhibited compromised corneal integrity associated with increased conjunctiva infiltrating CD4^+^ cells and reduced goblet cell hyperplasia, mice lacking either CCR6 or CXCR3 were found to have preserved corneal integrity alongside a suppressed CD4^+^ cell response and unaltered goblet cell density. Additionally, IL-17 and IFN-*γ* responses were high in cervical lymph nodes but suppressed in the ocular surface in the absence of both chemokine receptors (CCR6 and CXCR3). To test the reduced pathogenicity attributed to T cells lacking either of the chemokine receptors, adoptive transfer experiments were carried out in T cell-deficient RAG-1 mice. Lymphocytes harvested from DED mice were transferred into naïve RAG-1 recipient animals and disease assessed. Transfer of chemokine receptor-deficient T cells inhibited the infiltration of CD4^+^ T cells into the conjunctival epithelium and preserved goblet cell density. When inflammatory cytokine expression was assayed, authors found that mice transferred with chemokine receptor-deficient T cells displayed a clear inhibition of virtually all the cytokines (IL-6, IL-13, IL-17, and IFN-*γ*) and MMP 3 and 9 tested in corneal and conjunctival tissue [57].

Recently, it was identified that GM-CSF released by Th17 cells promotes recruitment and activation of CD11b^+^ myeloid cells in experimental DED. Analysis of cornea and conjunctiva tissues showed heightened mRNA expression and protein levels of GM-CSF upon DED induction. Intracellular flow cytometry confirmed double positive staining for IL-17 and GM-CSF. To test the putative role of GM-CSF on DED pathogenesis, CD11b^+^ myeloid naive cells were exposed to supernatants harvested from anti-CD3-stimulated purified CD4^+^ T cells from DED mice with or without anti-GM-CSF antibody. Only CD11b^+^ cells incubated with supernatants in the presence of isotype control antibody displayed significant high levels of MHCII and Ki67 antigens indicating activation and proliferation, respectively, as compared to those cells incubated with supernatants in the presence of anti-GM-CSF-neutralizing antibodies. When anti-GM-CSF treatment was given locally to assess the *in vivo* impact on myeloid cells during DED development, authors reported that the treatment caused diminished infiltration of CD11b^+^MHCII^hi^ cells into the ocular surface and low numbers of Th17 cells in draining lymph nodes paralleled with attenuated clinical signs as gauged by improved corneal integrity [58]. Also, IL-17 itself has been proven to be an important growth factor for B cells in the context of DED as recently described by Subbarayal et al. This study provided evidence that Th17 cells obtained from DED animal cervical lymph nodes had the ability to induce B cell proliferation and antibody switch in a contact-independent IL-17-dependent manner. Therefore, the pathogenic mechanisms displayed by Th17 may impact the humoral response by targeting B cells [59]. Evidence generated from different groups points out to a highly pathogenic role of infiltrating Th17 cells via its interaction with both innate and adaptive cells.

## 5. B Lymphocytes

B cells also contribute to the adaptive immune response. These cells are pivotal given their ability to secrete cytokines and as APCs; however, their key role as antibody-producing cells places them as central players in immune surveillance as well as in response to pathogen invasion. For ocular surface homeostasis, it has been reported that plasma cells are abundant in the human diffuse conjunctiva-associated lymphoid tissue (CALT) and the lacrimal drainage-associated lymphoid tissue (LDALT) and in the lacrimal glands [60]. Plasma cells residing in the ocular surface continuously release secretory IgA which is one of the most important humoral components in mucosal protection. IgA is thought to be responsible for limiting ocular microbiota invasion into deeper tissues [60]. The presence of B cells in human samples under sterile conditions is well documented; however, in contrast, mouse studies reported B cells (B220^+^CD3^−^) as one of the least prevalent cell populations in the ocular surface [48].

B cells have been studied in eye diseases from autoimmune origins including Sjögren's syndrome [61] and uveitis [62]. B cell depletion therapy using anti-CD20 antibodies has also been used as a treatment in eye diseases [63] suggesting an important role for B cells in the immunopathogenesis of several autoimmune diseases in the eye. However, the exact role of B cells in terms of either experimental or clinical DED is unclear. Although it has been reported that plasma cells reside in the human eye-associated lymphoid tissue (EALT) [60], one study of patients with DED found no change in the number of B cells; rather, it showed changes in CD4 and CD8 T cells and epithelial cell activation [64].

In terms of experimental DED, several reports have attempted to dissect the role of B cells. Stern et al. described that anti-kallikrein 13 antibodies arise upon desiccant stress-induced DED [65]. Also, when whole serum or purified IgG from DED mice were transferred into mice lacking T cells, this resulted in complement-dependent DED in recipient mice, suggesting activation of autoreactive B cells in donor mice [65]. To date, whether a late autoimmune response is triggered in human DED is unknown. Also, it was determined that mice naturally developing DED are a consequence of aging reflected by increased numbers of B cells in the lacrimal gland and severe ocular alterations [66]. Interestingly, it has also been found that by using the same DED mouse model (i.e., pharmacological inhibition of the lacrimal gland), no changes in B cell population were observed at 5 and 10 days upon DED induction while other intraepithelial cells like T cells and NK cells are more represented in this microenvironment [66].

More recently, Subbarayal et al. showed that IL-17, which has been described as a highly pathogenic cytokine in DED, can also target B cells leading to their proliferation. Furthermore, neutralizing IL-17 reduced germinal center formation and the pathogenicity of transferred B cells [59]. Therefore, it was shown that B cells can actively participate in promoting experimental DED.

Although B cells reside in the EALT, there is no clear consensus in regard to their specific participation in the immunopathology of DED. One can speculate that discrepancies between human and mouse DED mostly rely on the stage of DED, given that most of the mouse studies have addressed this in the context of acute DED (no more than 10 days postscopolamine administration) whereas human studies are conducted in individuals being affected for longer periods of time by DED.

## 6. The Unexplored Role of EALT-Resident Granulocytes in DED

Due to the contact between mucosal surfaces and the environment, certain immune cells are strategically positioned to rapidly respond to pathogenic threats. Early study of the ocular surface in rodents found that mast cells are present on the ocular surface and that their presence early in prenatal development may possibly contribute to the normal morphogenesis of corneal tissue [67, 68]. However, mast cells may also have a pathogenic role in allergic disorders, uveitis, and viral infections [69–71]. Although it has been shown that mast cells may play a dual role (i.e., contributing in corneal morphogenesis and promoting eye type 2 inflammatory reactions), no data yet exists for the possible role of mast cell populations on DED development.

Pioneering studies on resident leukocyte populations in the conjunctiva demonstrated that eosinophils are absent in the healthy eye, but during an allergic reaction, eosinophils can infiltrate the ocular surface and contribute to a Th2 inflammatory response [72]. Eosinophils have been widely studied in allergic ocular reactions including giant papillary conjunctivitis and vernal conjunctivitis where it was observed that these cells contribute to the inflammatory responses by releasing histamine and cationic proteins. In addition, corneal and conjunctival fibroblasts, endothelial cells, and mast cells have the ability to express eosinophil-attractant chemokines like eotaxin 1 [69, 73]. In the absence of basophils, there can be impaired infiltration of eosinophils into the ocular surface suggesting that basophils also promote eosinophil trafficking into the eye [74].

Although basophils have been poorly studied in the settings of inflammatory ocular disorders, recently, Sugita et al. reported an important role played by early-produced type 2 cytokines (i.e., IL-33 and thymic stromal lymphopoietin (TSLP)) but not IL-25 in promoting calpain-induced eye inflammation. The authors showed that ocular damage is attenuated in the absence of both IL-33 and TSLP, such phenomenon correlated with a low number of infiltrating basophils whose depletion also diminished eosinophil infiltration [74]. Thus, although little is known about the role of basophils, these findings put basophils as an additional disease-promoting innate cell population, in part, via modulating eosinophil responses.

Strikingly, the relevant role of innate cells like the abovementioned granulocytes in other inflammatory ocular diseases suggests that these cells are, most likely, also involved in generating tissue injury in response to desiccating stress, however, evidence still awaits to be shown.

## 7. Common Inflammatory Mediators in DED

Immune cell trafficking and activation employ soluble mediators that enable cells to infiltrate sites of tissue injury and modulate the immune response through receptor-ligand interactions. As immune system messengers, cytokines are key players in the stages of the inflammatory response. Examination of samples from patients with DED has revealed a pattern of inflammatory mediators associated with disease. Research groups have also identified a set of common mediators that can be detected in animal models of DED. A summary of these shared inflammatory mediators are shown in Table 1. Research to date demonstrates that Th1 and Th17 lymphocyte-associated mediators are predominant drivers in DED.

## 8. Future Directions

The eye contains numerous immune cells strategically positioned at the ocular surface with the primary goal of keeping the microenvironment tolerogenic to prevent unwanted tissue injury. In addition, resident immune cells also contribute to preserving the function of highly specialized cells involved in transduction of light stimuli into brain cells and conversion into images accomplishing the central function of the eye. Recently, reports have underscored the complex pattern of resident innate and adaptive immune cells interacting with ocular surface cells such as corneal and conjunctival epithelial cells, goblet cells, and stromal cells to maintain tear film integrity and corneal transparency which ultimately promotes ocular homeostasis. When ocular homeostasis breaks down, there is potential for the tolerogenic programs in resident cells to be altered and result in inflammatory processes that compromises both the tear film and ocular surface integrity. The triggers and mechanisms by which ocular tolerance disappears are starting to be understood, specifically in the context of DED.

The challenge to come is to better define the factors behind the onset of DED. Environmental factors such as exposure to temperature controlled air, low humidity, contact lens use, extended exposure to LED-based technologies, and recently diet and excessive antibiotic use are all being considered. Growth in our knowledge of the microbiome may also become a prominent area of study. Conjunctiva-resident bacteria have been linked to modulating DED, and efforts have been made to profile conjunctiva-resident microbiota under homeostatic conditions [75]. Starting to emerge is its potential as a biologic agent inhibiting pathogen proliferation due to colonization resistance [76]. Knowledge of bacterial communities promoting tolerance on ocular surfaces provides a potential new therapeutic alternative based on the success so far treating recurrent *C. difficile* infections and obesity [77]. Uncovering potential dysbiosis in ocular microbiota and the contribution to the pathogenesis of DED is required before implementation of novel therapies.

Future treatment strategies may also be developed based on existing therapeutic options for autoinflammatory diseases such as inflammatory bowel disease (IBD). In the case of IBD, application of cytokine-targeting therapies including anti-TNF-*α* therapy may have the potential to reduce DED clinical signs. Thus, identifying key cytokines driving ocular inflammation may guide the development of novel cytokine-based targeted therapies. Specifically, we recently found that the absence of macrophage migration inhibitory factor (MIF) protects against decreased tear volume and preserves tear's mucin patterns and goblet cell numbers in a scopolamine-induced DED mouse model (unpublished data). In addition, targeting nonimmune cells like goblet cells that are central in keeping tolerance through TGF*β* release and mucin production opens the possibility of exploiting goblet cell expansion as another way to attenuate ocular surface inflammation.

## 9. Concluding Remarks

We presented numerous studies describing the complex manner for how the eye is activated upon desiccating environmental stimuli and it also has the ability to further amplify such aggressive reactions observed in immunopathogenesis of experimental and human DED. Currently, anti-inflammatory drugs and artificial tears are the most often used treatment for DED demonstrating the importance of immune response during this disease. Unfortunately, the immunological landscape is not completely understood which limits the development of new therapeutic agents. Further, DED itself is a multifactorial idiopathic disease which makes it even more complicated to treat. New therapeutics directed at counteracting DED symptoms and deleterious effects caused by sustained inflammation on ocular surface are required.

In summary, inflammatory cellular infiltrate is a prominent characteristic of DED and it has been described that both innate and adaptive cells transmigrate and trigger DED. Continued examination is needed to reveal novel targets for development of effective therapeutics.

## Figures and Tables

**Table 1:** Cytokines correlated with ocular inflammatory DED.

Host	Infiltrated immune cells	Inflammatory mediators on the ocular surface	References
Human (15 NS-KCS patients)	CD4^+^ and CD8^+^	⬆ ICAM-1	17
Human (5 moderate DED patients)	CEC	⬆ nondifferences (IL-1*β*, IL-8, IL-6, and ICAM)	69
Human (30 dysfunctional tear syndrome patients)	ND	⬆ IL-6, IL-8, and TNF-*α*	3
Human (23 evaporative-type DED patients (46 eyes))	ND	⬆ IL-1Ra, IL-6, and CXCL/IL-8	70
Human (17 DED patients)	CD4^+^	⬆ CXCL9, 10, 11, and CXCR3	48
Human (35 aqueous-deficient dry eye DRY-aq patients, 36 lipid layer-deficient DRYlip patients, and 34 in combination of both (DRYaplip))	ND	⬆ IL-1*β*, IL-6, IL-8, TNF-*α*, and IFN-*γ*	71
Human (DED patients)	ND	⬆ IL-1*β*, IL-6, INF-*γ*, and TNF-*α*	72
Human (70 patients)	ND	⬆ IFN-*γ*	73
Human (34 DED patients with HIV infection and 32 DED patients without HIV infection)	ND	⬆ EGF, IFN-gamma-inducible protein 10 (IP-10, CXCL10)	74
Human (19 diabetic patients with DED and 15 nondiabetic patients with DED)	ND	⬆ IL-*β* and TNF-*α*	75
Human (15 samples in non-SS patients)	ND	⬆ IL-6, IL-17-A, and IL-23	76
Human (DED)	CD4^+^ and CD8^+^_RM_	⬆ CCR7^−^ T_EM_	49
Human (32 patients severe DED)	ND	⬆ IL-17 and TNF-*α*	77
Murine (BALB/c, T cell-deficient nude BALB/c (BALB/cByJHfH11<ʋ>), and C57BL/6)	Neutrophil, mononuclear cell, and CD4^+^	ND	22
Murine (C57Bl/6)	CD4^+^	⬆ IL-6, IL-17 mRNA	78
Murine (C57BL/6 desiccating stress)	CD4^+^IL-17^+^	⬆ TNF-*α*, IL-1*β*, IL-6, IFN-*γ*, IL-23, and IL-17A	53
Murine (C57BL/6)	CD11b^+^, CD4^+^IL-IFN-*γ*^+^, and CD4^+^IL-17^+^	⬆ CCR6, CCL20, and IL-17A	54
Murine	CD11b^+^ and CD4^+^IL-17^+^	⬆ IL-17A and GM-CSF	56
Murine (C57BL/6)	CD4^+^CXCR3^+^ T cells	⬆ IL-1*β*, Il-6, TNF-*α*, IFN-*γ*, and ROS	79

DED: dry eye disease; ND: nondetermined; Mo: macrophages; non-SS: non-Sjögren syndrome; EGF: epidermal growth factor and IFN-gamma-inducible protein 10 (IP-10, CXCL10); CEC: conjunctival epithelial cells; NS-KCS: non-Sjögren's syndrome keratoconjunctivitis sicca; T_RM_: resident memory T cells, T_EM_: effector memory T cells; ROS: reactive oxygen species.
